# Molecular Cloning and Functional Identification of a Squalene Synthase Encoding Gene from Alfalfa (*Medicago sativa* L.)

**DOI:** 10.3390/ijms20184499

**Published:** 2019-09-11

**Authors:** Junmei Kang, Qiaoyan Zhang, Xu Jiang, Tiejun Zhang, Ruicai Long, Qingchuan Yang, Zhen Wang

**Affiliations:** Institute of Animal Science, Chinese Academy of Agricultural Sciences, Beijing 100193, China; kangjunmei@caas.cn (J.K.); zhqynancy@126.com (Q.Z.); jiangxu2009@yeah.net (X.J.); tiejunzhang@126.com (T.Z.); dragongodsgod@163.com (R.L.); qchyang66@163.com (Q.Y.)

**Keywords:** alfalfa, saponins, squalene synthase, transgenic alfalfa

## Abstract

The quality of alfalfa, a main legume forage worldwide, is of great importance for the dairy industry and is affected by the content of triterpene saponins. These natural terpenoid products of triterpene aglycones are catalyzed by squalene synthase (SQS), a highly conserved enzyme present in eukaryotes. However, there is scare information on alfalfa *SQS*. Here, an open reading frame (ORF) of *SQS* was cloned from alfalfa. Sequence analysis showed *MsSQS* had the same exon/intron composition and shared high homology with its orthologs. Bioinformatic analysis revealed the deduced MsSQS had two transmembrane domains. When transiently expressed, GFP-MsSQS fusion protein was localized on the plasma membrane of onion epidermal cells. Removal of the C-terminal transmembrane domain of MsSQS improved solubility in *Escherichia coli*. *MsSQS* was preferably expressed in roots, followed by leaves and stems. MeJA treatment induced *MsSQS* expression and increased the content of total saponins. Overexpression of *MsSQS* in alfalfa led to the accumulation of total saponins, suggesting a correlation between *MsSQS* expression level with saponins content. Therefore, *MsSQS* is a canonical squalene synthase and contributes to saponin synthesis in alfalfa. This study provides a key candidate gene for genetic manipulation of the synthesis of triterpene saponins, which impact both plant and animal health.

## 1. Introduction

In model legume *Medicago truncatula*, the triterpene saponins, important terpenoid natural products, are glycosides of at least five different triterpene aglycones catalyzed by squalene synthase (SQS/SS), squalene epoxidase (SE) and beta-amyrin synthase (beta-AS) [1]. Among them, SQS, the key enzyme of the saponin biosynthesis pathway, serves as a potential adjusting point managing carbon flux from isoprenoids biosynthetic pathway into triterpene and sterol biosynthesis [2]. SQS is a structural conservation enzyme present in fungi, animals and plants [3,4,5]. The membrane-bound enzyme binds to the endoplasmic reticulum and catalyzes the bicondensation of two identical molecules of farnesyl diphosphate (FPP) to squalene, the precursor of sterols and triterpenoid [2,6]. In higher plant, SQS encoding genes have been identified from a wide range of species, including model plants (e.g., *Arabidopsis* and barrel clover) [1,7], crops (e.g., rice, soybean, barley and potato) [8,9,10,11], the economically or pharmaceutically important plants (e.g., tobacco and ginseng) [12,13] and trees [14,15]. Most of these *SQS* genes have been characterized by bioinformatics approaches to evaluate the physicochemical properties and structural characteristics, and some were explored by molecular techniques to analyze their biological functions.

An increasing body of evidence has shown that *SQS* genes expressed ubiquitously in plant organs with varying levels are functionally conserved. On one hand, the triterpenoid biosynthesis was reported to be strongly related with the expression level of the *SQS* transgene in medicinal plants including ginseng, *Eleutherococcus senticosus*, *Euphorbia tirucalli*, *Bupleurum falcatum* and *Withania somnifera* [16,17,18,19,20]. For example, overexpression of *PgSS1*, a *Panax ginseng* squalene synthase, in the adventitious roots of the transgenic ginseng resulted in an enhanced activity of PgSS1 enzyme and a remarkably increased content of both phytosterols and ginsenoside [18], indicating that PgSS1 is a key regulatory enzyme for the biosynthesis of phytosterols and triterpene saponins. On the other hand, complementation of yeast *erg9* mutant strain 2C1, which is a squalene synthase-deficient mutant lacking SQS activity [21], provided direct evidence that despite the relatively low sequence homology to yeast SQS, these plant SQSs share the conserved function of squalene synthase with its ortholog in fungi. For example, both *PgSS2* and *PgSS3* restored ergosterol prototrophy of the *erg9* mutant [17], suggesting their activity in squalene biosynthesis. Nguyen et al. [11] found that overexpression of either *GmSQS1* or *GmSQS2* resulted in slow growth of *erg9* in the medium lacking ergosterol, indicating a partial complementation of the squalene synthase activity by the two soybean *SQSs*. For the two *Arabidopsis SQS* genes (*AtSQS1* and *AsSQS2*), which are organized in a tandem array, *AtSQS1* was reported to be widely expressed in most tissues throughout plant development, while the expression of *AsSQS2* was confined to the hypocotyl, the vascular tissue of leaf and cotyledon petioles. Interestingly, the former *SQS*, but not the latter one, was reported to be able to confer ergosterol prototrophy to *erg9* mutant strain [22]. Consistently, upon the exposure of tobacco cell suspensions to the SQS specific inhibitor squalestatin, a rapid decrease in SQS activity and a parallel accumulation of its substrate farnesol were detected [23]. These findings indicated that *SQS* plays an important role in regulating the triterpene biosynthetic pathway.

Alfalfa (*Medicago sativa* L.), a major legume forage worldwide, is one of the most valuable legume plants with high protein content. The legume forage possesses a wide range of secondary metabolites including triterpene saponins. Alfalfa saponins (ASs) are pentacyclic triterpene and these compounds occur as glycosides of several aglycones. The biological activities of saponins depend on the aglycone structure and the composition of the carbohydrate side-chains [24]. In recent years, 55 triterpene saponins have been found in alfalfa [25] and some have been demonstrated to have promising activities for pharmacological applications, including antioxidant, anti-inflammatory and anticancer activities [26,27,28]. From the nutritional point of view, the saponin activities, such as foaming properties, hemolytic and antimicrobial properties, throat-irritating effects and modulatory effects on the permeability of the intestinal membrane, are of the greatest importance because these features affect microbial fermentation and the digestion efficiency of alfalfa [29]. These unfavorable effects have restricted the optimum use of alfalfa in animal feed. On the other hand, ASs were found to negatively affect the development of spotted alfalfa aphid and were effective to control rice blast by preventing the fungal attack of several rice cultivars [30,31]. Hence, investigation of the biosynthesis pathway of saponins may facilitate the regulation of saponin production at appropriate levels in alfalfa, which would benefit the health of both animal and plant. However, little is known about alfalfa SQS, the key early enzyme of triterpene aglycone formation. In this study, we cloned and characterized *MsSQS* from alfalfa. Our results demonstrated that *MsSQS* is a canonical squalene synthase encoding gene preferentially expressed in roots. *MsSQS* is MeJA inducible and overexpression of *MsSQS* increased the amount of saponins in the transgenic alfalfa plants, implying the involvement of *Ms*SQS in saponin synthesis.

## 2. Results

### 2.1. MsSQS Encodes a Potential Squalene Synthase with High Sequence Identity to SQS Orthologs in Higher Plant

**Figure 1:**
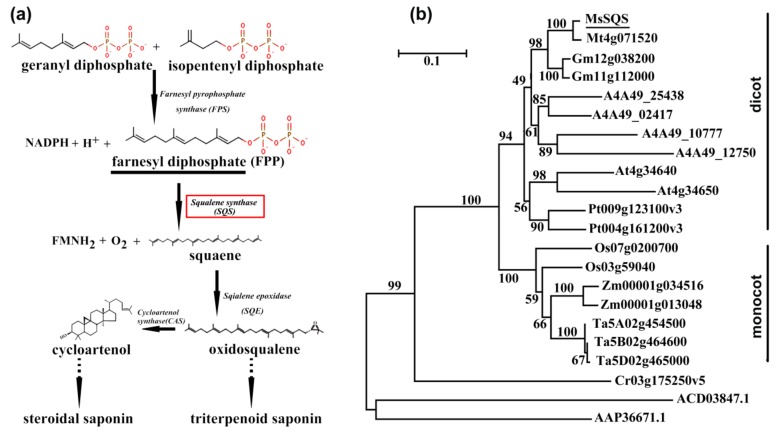
Illustration of the synthesis pathway of triterpene saponins and phylogenetic analysis of SQS proteins from the indicated species. (**a**) Illustration of the major saponins biosynthesis pathway in plants. Squalene synthase (*SQS*) is boxed in red. (**b**) The predicted polypeptide of SQS from the indicated species was analyzed using MEGA (version 6) [32]. The bar represents the evolutionary distance and MsSQS was underlined. Gene accession numbers based on EnsemblPlants (http://plants.ensembl.org/index.html) are as follows: *Arabidopsis thaliana* (At): At4g34640, At4g34650; *Chlamydomonas reinhardtii* (Cr): Cr03g175250v5; *Glycine max* (Gm): Gm12g038200, Gm11g112000; *Homo sapiens*: AAP36671.1; *Medicago truncatula* (Mt): Mt4g071520; *Nicotiana tabacum*: A4A49_02417, A4A49_25438, A4A49_10777, A4A49_12750; *Oryza sativa* (Os): Os07g0200700, Os03g59040; *Populus trichocarpa* (Pt): Pt009g123100v3, Pt004g161200v3; *Saccharomyces Cerevisiae*: ACD03847.1; *Triticum aestivum* (Ta): Ta5A02g454500, Ta5B02g464600, Ta5D02g465000, and *Zea mays* (Zm): Zm00001g013048, Zm00001d034516.

Arabidopsis genome encodes two squalene synthase (SQS) proteins (SQS1 and SQS2) with 78.5% sequence identity [22]. To identify *SQS* orthologs in legume forage alfalfa, the model legume *Medicago truncatula* genome database (http://plants.ensembl.org/Medicago_truncatula) was referred. Using AtSQS1, the only functional Arabidopsis SQS [22], as a query sequence, our BLAST search against the most updated *M. truncatula* genome database hit one gene (Mt4g071520) annotated as squalene synthase with a homology of 79.0% (Table S1). To clone *SQS* gene from alfalfa by RT-PCR, degenerate primers were designed based on the open reading frame (ORF) of *SQS* from *Arabidopsis* and *M. truncatula* (primers are listed in Table S2). A fragment of 1439 bp was amplified and sequence analysis predicted an ORF of 1242 bp encoding a polypeptide of 413 amino acids (Figure S1). The estimated molecular weight of the predicted enzyme is about 47.25 kDa with a theoretical isoelectric point of 7.53 (Table S3). Protein BLAST search demonstrated that it encodes squalene synthase, which converts two molecules of farnesyl diphosphate (FPP) into squalene via an intermediate: presqualene diphosphate (PSPP) (Figure 1a). Thus, it was designated as *MsSQS*, a squalene synthase encoding gene first identified from forage crop alfalfa.

Sequence search for *SQS* homologus genes in several plant species demonstrated that compared with yeast or animals which have single *SQS*, these plants, except *M. truncatula*, possess a couple of *SQSs* (Table S3), suggesting an expansion of *SQS* family members in these plant species. Phylogenetic analysis showed that SQSs from higher plant, algae, fungi and human were clustered into separate branches individually (Figure 1b), suggesting a relatively far evolution distance from one another. Higher plant SQS enzymes were split into two main branches: monocot and dicot. As expected, MsSQS was grouped into the dicot branch containing soybean, tobacco, populus and barrel clover, and the SQS proteins from rice, wheat and maize were grouped into the monocot branch (Figure 1b). Consistently, sequence homology analysis revealed that MsSQS is about 57.0%, 76.8%, 79.0%, 92.0% and 97.8% identical to the overall polypeptides of *Chlamydomonas*, rice, *Arabidopsis*, soybean and barrel clover, respectively, while the sequence identity to yeast and human SQS enzyme is about 41.4% and 48.7%, respectively (Table S1). These results indicated that relative to SQS enzymes in yeast and human, MsSQS shared a higher identity with its orthologs from a variety of plant species ranging from green alga to barrel clover. Hence, the cloned alfalfa *SQS* encodes a squalene synthase highly identical to its ortholog in *M. truncatula*.

### 2.2. MsSQS Is a Canonical Squalene Synthase with the Common Features of SQS

**Figure 2:**
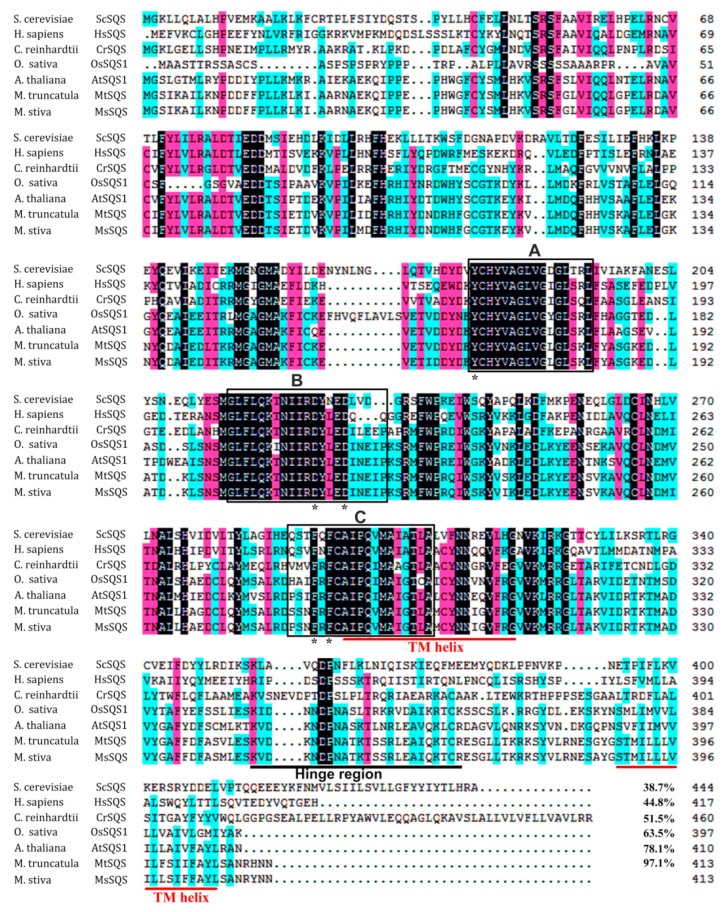
Sequence alignment of the representative squalene synthase proteins from the indicated species. Gene accession numbers: ACD03847.1 (*S. Cerevisiae*); AAP36671.1 (*H. sapiens*); Cr03g175250v5 (*C. reinhardtii*); Os07g0200700 (*O. sativa*); At4g34640 (*A. thaliana);* Mt4g071520 (*M. truncatula*). Sequence was aligned using DNAMAN (Version 7) (Lynnon Corporation, Quebec, Canada). Homology level was highlighted by shading in color: black for 100%, pink for ≥ 75% and blue for ≥ 50% identity. Conserved segments (A, B and C) were boxed, the predicted transmembrane helix and the hinge region with low similarity to yeast was underlined in red and in black, respectively. An asterisk (*) indicates the conserved key residues in the three segments.

To determine the exon–intron composition of *MsSQS*, the genomic sequence was amplified and assembled (Figure S2). Our analysis revealed that like most of its orthologs from higher plant (18 out of the 19 *SQS* genes), *MsSQS* was composed of 13 exons, ten of which (exons 2–11) individually shared an identical length in size. In contrast, the gene structure of *SQS* from human and *Chlamydomonus* differed from that of higher plant (Table 1). Comparison of 22 *SQS* genes from 12 species including human, yeast, green algae and higher plant demonstrated that a vast majority of these *SQS* transcripts encoded proteins consisting of 401–413 amino acids (Table S3). The domains (66–392 a.a. in the case of AtSQS1) of the three functional segments, namely A, B and C (Figure 2), are encoded by the remarkably conserved exons, and are considered important for binding, catalysis and regulation of SQS-type enzymes [33].

**Figure 3:**
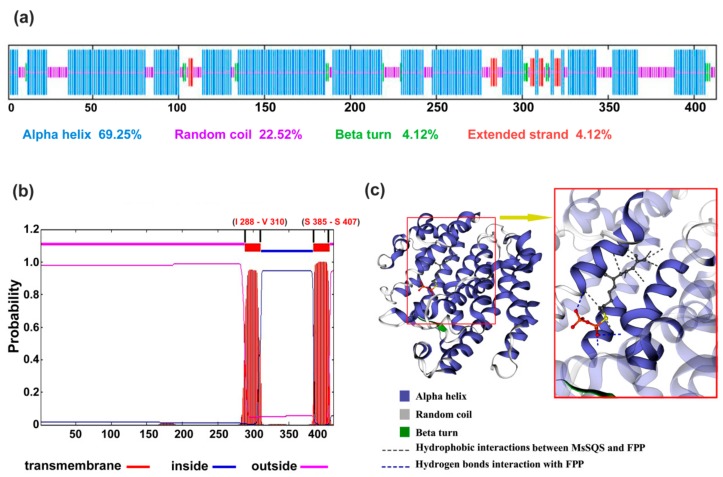
Analysis of the secondary structure and predicted 3-D model of MsSQS. (**a**) Annotation of the secondary structure of MsSQS using SOPMA online server (https://npsa-prabi.ibcp.fr/cgi-bin/npsa_automat.pl?page=npsa_sopma.html). Components of the secondary structure were listed in color corresponding to the individual structure. (**b**) The predicted transmembrane regions of MsSQS using TMHMM Server (v. 2.0) (http://www.cbs.dtu.dk/services/TMHMM/). (**c**) The homology-based 3-D structure of MsSQS generated with SWISS-MODEL (left panel). The center channel (boxed in red) of MsSQS was enlarged showing the interaction with farnesyl diphosphate (FPP) (right panel).

The secondary structure prediction showed that the main components of MsSQS are the alpha helix (69.25%) and the random coil (22.52%) (Figure 3a). A similar structure was predicted for SQS proteins from other species with the alpha helix ranging from 66.17% to 71.04%, and the random coil from 18.03% to 24.92% (Table S4). Scanning transmembrance protein topology using TMHHM software [34] showed that MsSQS possesses two transmembrane helix domains composed of 23 amino acid residues each: Helix I is from Ile (I) 288 to Val (V) 310, and Helix II from Ser (S) 385 to Ser (S) 407 (Figure 3b). The location of the two transmembrane domains is in agreement with the findings of other legume species such as soybean, barrel clover and *Lotus japonicus* [1,6,11]. The C-terminal transmembrane domain is highly hydrophobic with low sequence similarity (Figure 2), and is presumably involved in the function of membrane targeting and anchoring [5].

The 3-D structure of MsSQS was predicted using structure modeling on the Swiss model server (https://www.swissmodel.expasy.org) and human SQS with 44.8% sequence identity served as template for comparative modeling. The predicted structure of MsSQS consisting predominantly of alpha helices is folded as a single domain with a large channel running through the center surrounded by helices (Figure 3c), a typical structure of some isoprenoid biosynthetic enzymes [5]. Substrate prediction targeted the farnesyl pyrophosphate (FPP), the substance catalyzed by SQS to produce squalene, in the center channel of MsSQS with hydrogen bonds and hydrophobic interactions (Figure 3c). Hence, MsSQS not only has the same exon/intron composition but also possesses the conserved functional domains shared by a wide range of plants.

### 2.3. The MsSQS-GFP Recombinant Protein Resided Transiently on the Plasma Membrane of Onion Epidermal Cells

**Figure 4:**
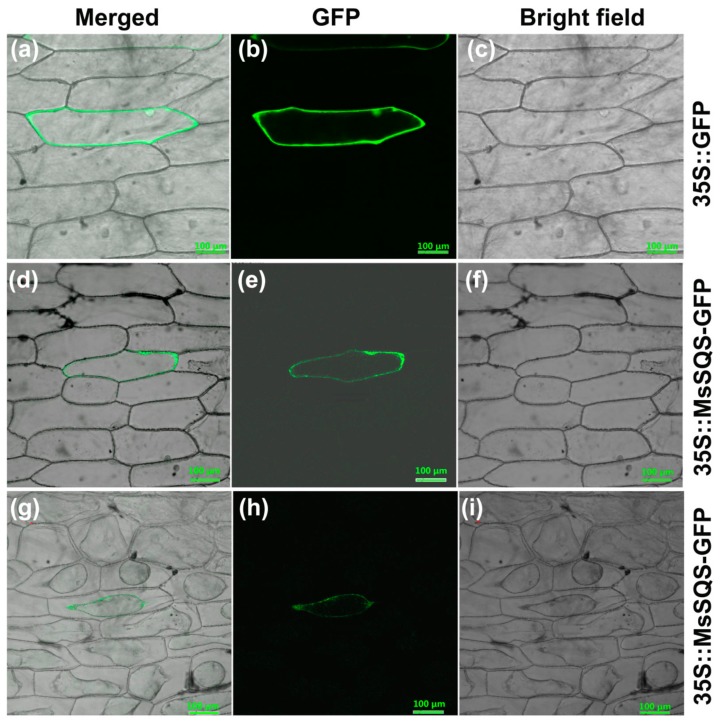
Subcellular localization of the 35S::MsSQS-GFP in onion epidermal cells. The *MsSQS-GFP* fusion construct and control vector pA7-GFP [35] were individually transformed into onion epidermal cells by microprojectile bombardment. Image was captured using a confocal laser scanning microscope (Olympus FV500). (**a**–**c**) Images of onion epidermal cells expressing *35S::GFP* taken under GFP fluorescence (**b**) or in bright field (**c**), the merged one was shown in (**a**); (**d**–**f**) Images of onion epidermal cells expressing *35S::MsSQS-GFP* taken under GFP fluorescence (**e**) or in bright field (**f**), the merged one was shown in (**d**); (**g**–**i**) Images of sucrose-treated (30%) onion epidermal cells expressing *35S::MsSQS-GFP* taken under GFP fluorescence (**h**) or in bright field (**i**), the merged image was shown in (**g**). Bar = 100 µm.

In *Arabidopsis*, SQS1 and SQS2 were predicted to localize in endoplasmic reticulum membrane and plasma membrane (https://www.arabidopsis.org/). To examine the subcellular localization of MsSQS, *MsSQS-GFP* driven by the 35S promoter was transformed into onion epidermal cells by microprojectile bombardment with *35S::GFP* as positive control. As shown in Figure 4, the expression of GFP control was distributed mainly in both nucleus and plasma membrane of the onion epidermal cell (Figure 4a–c), whereas the MsSQS-GFP fusion protein was observed on the plasma membrane (d–f). To confirm its membrane residence, cells transiently expressing *35S::MsSQS-GFP* were exposed to sucrose solution (30%), and images were captured after plasma membrane separated from cell wall due to the water loss. As shown in Figure 4g–i, the green fluorescence was observed on an irregularly-shaped membrane caused by sucrose treatment, indicating that MsSQS-GFP was localized on the plasma membrane.

### 2.4. The Transmembrane Domain Affected the Solubility of MsSQS Expressed in Escherichia coli

**Figure 5:**
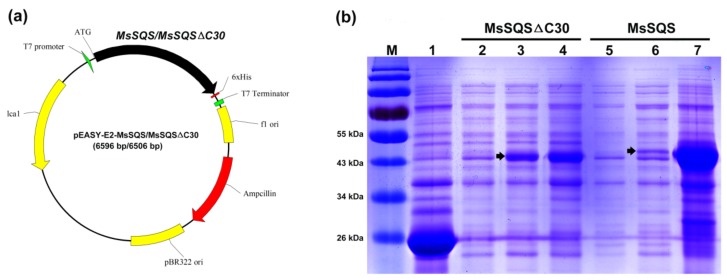
Vector construct and expression analysis of MsSQS/MsSQSΔC30 by SDS-PAGE. (**a**) Construct of pEASY-E2-MsSQS or pEASY-E2- MsSQSΔC30; (**b**) SDS-PAGE analysis of MsSQS and MsSQSΔC30 expressed in *E. coli.* M: protein ladder, Lane 1: total proteins from IPTG-induced cells expressing pEASY-E2 (Control), Lane 2–Lane 4: proteins from cells expressing pEASY-E2-MsSQSΔC30, for total proteins without IPTG treatment (Lane 2), proteins from the supernatant (Lane 3) or from the total cells (Lane 4) treated by IPTG; Lane 5–Lane 7: proteins from cells expressing pEASY-E2-MsSQS, for total proteins without IPTG treatment (Lane 5), proteins from the supernatant (Lane 6) or from the total cells (Lane 7) treated by IPTG.

Recent analyses of SQS enzymes from several plant species have shown that SQS proteins have two transmembrane regions in the carboxy-terminal [3,36], suggesting they are membrane proteins residing on endoplasmic reticulum membrane and plasma membrane. To compare the protein solubility, a truncated (1–383 a.a.) protein lacking the last 30 amino acids (MsSQSΔC30), which includes the C-terminal transmembrane domain, was produced. The peptide deletion of MsSQSΔC30 resulted in about 3.4 kDa less molecular weight than MsSQS (47.25 kDa). Both *MsSQS* and *MsSQSΔC30* were separately subcloned into vector pEASY-E2 and expressed in *E. coli* (DE3) (Figure 5a). Cells expressing the individual construct were induced with 0.8 mM IPTG at 30 °C for 5 h and resuspended in the extraction buffer for SDS-PAGE analysis. Figure 5b demonstrated that compared with MsSQS from the supernatant, the expression of the truncated SQS (MsSQSΔC30) was clearly enriched in the supernatant (Lane 3 vs. Lane 6). In contrast, MsSQS was intensively expressed from total cell extract (Lane 7). These results suggested that removal of the C-terminal transmembrane region improved the solubility of the membrane protein MsSQS.

### 2.5. Expression Analysis of MsSQS in Alfalfa Tissues and under MeJA Treatment

**Figure 6:**
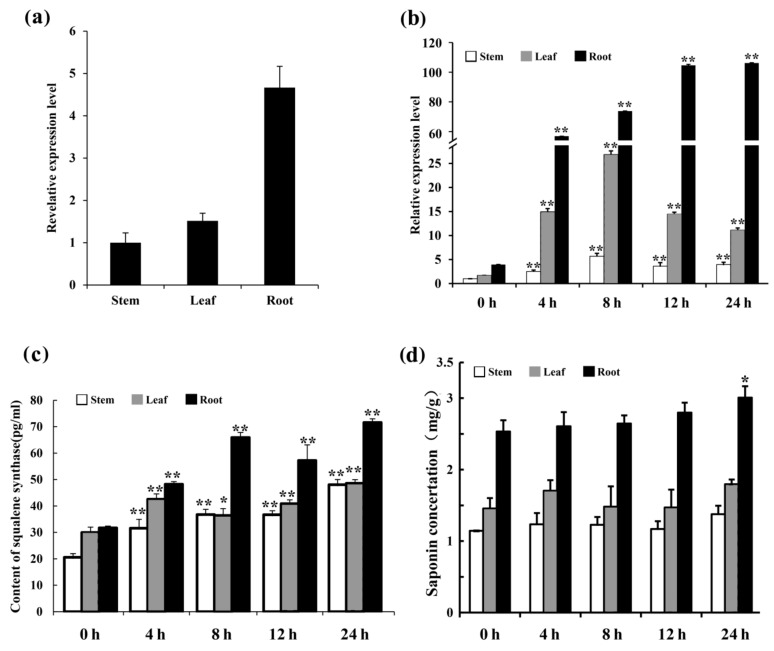
Analysis of *MsSQS* expression, enzyme content, or saponin concentation in alfalfa tissues treated by MeJA. (**a**) The relative expression of *MsSQS* in alfalfa tissues by qRT-PCR. Thirty-day-old seedlings were used for the analysis. Data were normalized to stem; (**b**) The relative expression of *MsSQS* in the indicated alfalfa tissues treated by MeJA. Data were normalized to stem at 0 h. Thirty-day-old seedlings were treated with MeJA (200 μM) for the indicated time points, and plants grown in Hoagland’s solution with the same amount of ethanol in 200 μM MeJA was used as control (0 h). Alfalfa *Actin* gene was used as internal control; (**c**) content of the MsSQS enzyme (pg/mL) in the alfalfa tissues used in (**b**); (**d**) concentration (mg/g) of total saponins in the tissues (dry weight) used in (**b**). Bars represent the mean ± SD of three biological replicates. * and ** indicate *p* < 0.05 and *p* < 0.01, respectively (Student’s t-test).

In order to determine the expression pattern of *MsSQS* in alfalfa tissues, qRT-PCR was performed. As shown in Figure 6a, *MsSQS* was detected in stems, leaves and roots. Relatively, the expression level in leaves and roots was about 1.5- and 4.8-times of that in stems (Figure 6a), suggesting that *MsSQS* was preferentially expressed in root tissues. The result is consistent with the observations that *SQS* was predominantly expressed in roots of soybean and *Tripterygium wilfordii* [11,37].

An increasing body of evidence has shown that metyl jasmonate (MeJA) treatment induced *SQS* transcript level in several plants [15,17,18]. We tested the expression of *MsSQS* in stem, leafand root under MeJA (200 μM) treatment by qRT-PCR. Our results demonstrated that *MsSQS* transcript in root was rapidly increased at 4 h and the induction was progressively enhanced by the treatment, while *MsSQS* in stem and leaf reached a summit at 8 h and decreased to about three-fold and five-fold of the control, respectively at 24 h (Figure 6b). The significant up-regulation of *MsSQS* by MeJA treatment indicated that *MsSQS* is MeJA-inducible. Measurement of the content of squalene synthase showed that the enzyme was progressively increased by MeJA treatment in the three tissues (Figure 6c). Consequently, a relatively higher content of total saponins was detected with root saponins significantly accumulated at 24 h time point (Figure 6d). These results suggested that *MsSQS* was a MeJA-inducible gene and its transcript was correlated with the content of saponins in alfalfa.

### 2.6. Overexpression of MsSQS Increased the Content of Saponins in Transgenic Alfalfa

**Figure 7:**
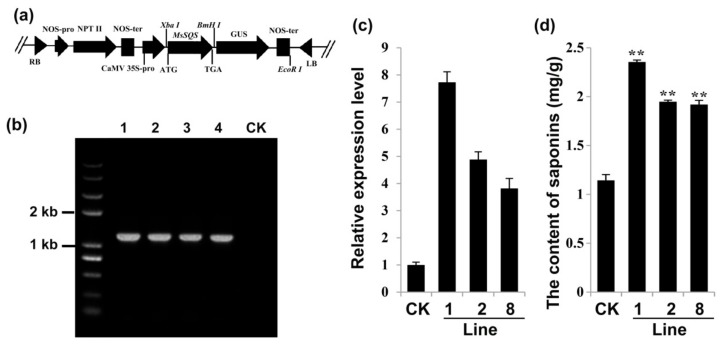
Generation of the transgenic alfalfa harboring *35S::MsSQS* construct and analysis of *MsSQS* level and saponin content in *MsSQS-*overexpression alfalfa plants. (**a**) Illustration of the *MsSQS* overexpression vector with *Xba* I and *Bam* HI used to subclone *MsSQS* into pBI-121; (**b**) Verification of the *35S::MsSQS* construct in alfalfa plants resistant to kanamycin by PCR using plasmid or genomic DNA as template (Lane 1–4); Lane 1: plasmid harboring *35S::MsSQS* was used as the positive control, Lane 2–4: three independent transgenic alfalfa lines (Line 1, Line 2 and Line 8, respectively), CK: transgenic alfalfa expressing pBI-121 (the negative control). (**c**) Analysis of the relative expression level of *MsSQS* in leaves by qRT-PCR in the three representative transgenic lines. The transgenic alfalfa expressing pBI-121 was used as the control; (**d**) Content of total saponins (mg/g) in leaves (dry weight) of the indicated transgenic lines. Bars represent the mean ± SD of three biological replicates. ** indicates *p* < 0.01 (Student’s t-test).

To investigate the function of *MsSQS* in saponin biosynthesis, the ORF of *MsSQS* was subcloned into pBI-121 (Figure 7a) and introduced into alfalfa plants via Agrobacterium-mediated transformation [38]. The representative kanamycin-resistant plants were verified by PCR with genomic DNA as template. As shown in Figure 7b, a fragment covering the 35S promoter and *MsSQS* was amplified in the transgenic lines, as well as from the plasmid itself (positive control), indicating the successful integration of the *35S::MsSQS* construct into the alfalfa genome. Expectedly, no amplification was detected in either the transgenic alfalfa expressing pBI-121 vector or the non-transgenic plant (Figure 7b). The transcriptional analysis by quantitative RT-PCR demonstrated that the level of *MsSQS* of the three representative lines (Lines 1, 2 and 8) was increased to 7.7-, 4.9-, and 3.8-fold of the line expressing pBI-121 vector, respectively (Figure 7c), suggesting an enhanced expression in the 35S::*MsSQS*expressing transgenic lines relative to the control plants.

The overexpression plants exhibited no abnormal phenotype compared to the non-transgenic ones (Figure S3). Since squalene synthase is one of the enzymes that catalyze the formation of triterpene saponins, we measured the content of saponins in the transgenic alfalfa. Figure 7d showed that compared with the control which contained 1.2 mg/g of saponins, the saponins amount in the transgenic line 8, Line 2 and Line 1 was about 1.9 mg/g, 2.0 mg/g and 2.4 mg/g, respectively. These results indicated that the content of saponins in the transgenic alfalfa was almost doubled, suggesting that the synthesis of saponins was associated with the transcriptional level of *MsSQS*.

## 3. Discussion

The saponins are naturally occurring surface-active glycosides, which include steroid and triterpenoid glycosides in a great deal of plant species, and compared with steroidal saponins which are mainly found in moncotyledons, triterpene saponins are generally predominant in dicotyledons [39]. Due to the potential applications of triterpenoid saponins in food and pharmaceutical industries, legumes, such as soybeans and peas, which serve as main dietary sources, are extensively studied [40,41]. This study focused on squalene synthase (SQS), one of the early enzymes in saponin synthesis pathway, in legume forage alfalfa, which is the main non-food source of saponins. Our findings provided strong evidence that the membrane protein MsSQS belonged to the highly conserved SQS family with enzymatic features, and that in the transgenic alfalfa constitutively expressing *MsSQS*, the content of saponins is associated with *MsSQS* level.

Based on sequence analysis, an increasing number of *SQS* genes have been identified from a wide range of eukaryotic species especially plants of medicinal importance [36,42]. In agreement with the phylogenetic analysis showing plant SQSs were grouped separately from the subclass of yeast or human [8], our analysis revealed that plant SQS proteins had higher sequence homology (Figure 1b, Table S1), suggesting a closer evolutionary distance within the plant kingdom relative to the non-plant species. The notion is supported in part by our observation that in higher plant, *SQS* genes have a universal pattern of exon/intron composition with 13 exons each (Table 1). Among them, 76.9% (10/13) of the exons except the first and the last two, are individually at the same length for the eight plants. In contrast, *SQS* in green algae and human contains 11 and nine exons, respectively, and the length of individual exons is different from the corresponding ones in higher plant. These findings suggest that *SQS* in higher plant shares remarkably conserved exon/intron boundaries.

Different from the gene composition of *SQS*, which is conserved within higher plants, the overall architecture of SQS enzyme has been reported to be highly identical in eukaryotes [5]. Indeed, MsSQS, together with its eukaryotic homologs, shared the conserved functional domains with specific amino acid residue(s) at certain site(s). First, our analysis of the deduced peptides for squalene synthases highlighted alpha helix and random coil as the main components of SQS secondary structure (Figure 3a, Table S4). On average, the alpha helix accounts for about 68.66% and the random coil 22.00% of the peptides, respectively. Recent studies have found more plants, such as several ginseng species and Fabaceae family plants [3,13] with a similar secondary structure. Secondly, SQS proteins share three conserved domains (A, B and C) and certain amino acid residues within these domains are essential for catalysis as reported in rat [33]. These residues are present in higher plant including *Arabidopsis*, soybean and barrel clover [1,11,22]. For segment A, Tyr (Y) 168 is presumably involved in the first step of catalysis, and the Asp-rich motif (DXXXD) of segment B is considered to be the active center for substrate binding with the presence of Mg^2+^. The two Phe (F) 283 and F 285 of segment C may contribute to the second-step catalysis [4,43]. Thirdly, the alpha helices of the monomeric SQS protein form a cave-like active center and transmembrane domain(s) at the C-terminus. In recent years, the transmembrane regions have been identified by bioinformatics approaches in a variety of species, such as *Siraitia grosvenorii*, wintersweets and Cucurbitaceae family plants [36,44,45]. The enzymatically active center folded by helices supplies an interacting surface with SQS substrate FPP via hydrogen bonds and hydrophobic interactions (Figure 3). Taken together, *MsSQS* identified from alfalfa encodes a canonical squalene synthase sharing identical gene structure and highly conserved functional domains with its orthologs in higher plant.

It appears that membrane enzyme squalene synthase encoding gene *MsSQS* affects the content of saponins in alfalfa. Expression analysis indicated that the ubiquitous *MsSQS* was expressed preferentially in roots (Figure 6). The root-preferred pattern was observed for *GmSQS1* in soybean [11], *SgSQS* in *Siraitia grosvenorii* [44], *HsSQS1* in *Huperzia serrata* [46] and *TwSQS* in traditional Chinese medicinal plant *Tripterygium wilfordii* [37]. Some plants, such as *Withania somnifera* [47], *Betula platyphylla* [15] and *Arabidopsis* [22], displayed a leaf-predominant pattern, suggesting that the spatial and temporal expression patterns of *SQS* genes vary greatly in different plants. Consistent with the observations that the *SQS* transcript was activated by MeJA induction [17,18,37], *MsSQS* was up-regulated upon exposure to MeJA and the stimulation resulted in an increased amount of the MsSQS enzyme (Figure 6). It has been reported that the hydrophobic amino acid residues at the C-terminal of SQS contribute to the membrane anchoring function [4,5]. Deletion of the transmembrane domain enhanced the solubility of MsSQS, as well as the recombinant SQS proteins from several species [37,48], and the truncated SQS was capable of converting FPP to form squalene, indicating the folding capability and the catalytic activity remained unchanged [45,49]. Interestingly, fungal squalene synthases have a unique hinge region (26 amino acid residues) linking the catalytic and membrane-spanning domains, and the hinge domain is essential for functional SQS in yeast but not for animals or plants [4]. We showed that overexpression of *MsSQS* in alfalfa significantly increased the content of total saponins in the transgenic plants. The correlation coefficient between *MsSQS* expression level and saponins content is 0.978, indicating that the amount of saponins in the transgenic alfalfa is strongly correlated with the transcriptional level of *MsSQS*. Therefore, our study provides evidence that *MsSQS* encodes a typical squalene synthase and is positively involved in the synthesis of saponins. Future work is to investigate the enzymatic activity of *MsSQS* and the biological functions using the *SQS* mutant from model plants.

## 4. Materials and Methods

### 4.1. Plant Materials and Growth Conditions

*Medicago sativa* cv. Zhongmu No. 1 bred by our lab (the Institute of Animal Science, the Chinese Academy of Agricultural Sciences), was used in the study. Seeds were germinated in regular soil (pot in diameter of 20 cm) or Hoagland’s solution in growth chamber at 21 °C with 14 h light/10 h dark.

### 4.2. Plant Treatment

Alfalfa seeds were germinated and grown in Hoagland’s solution. For expression analysis in plant tissues, leaves, stems and roots from 30-day-old hydroponic seedlings were collected separately and frozen in liquid nitrogen. For hormone treatment, at day 30, half of the plants were transferred into freshly prepared regular Hoagland’s solution, and the other half into fresh Hoagland’s solution supplemented with MeJA (200 μM). Treatment of 2, 4, 8, 12 and 24 h was conducted, and tissues from the treated seedlings t and non-treatment were harvested separately at the individual time point. Plant samples were frozen in liquid nitrogen for further analysis.

### 4.3. Cloning of MsSQS from Alfalfa and Expression Analysis by Quantitative Real-Time PCR

Genomic sequence was amplified by nested PCR and assembled using DNAMAN. Total RNA was extracted from alfalfa using Trizol reagent. RNA concentration was determined with a NanoDrop 2000 spectrophotometer (Thermo Scientific, Santa Cruz, CA, USA). One μg of total RNA was used for the first-strand cDNA synthesis using the PrimeScript^TM^ 1st strand cDNA Synthesis Kit (Takara Biomedical Technology Corporation, Beijing, China). Degenerate primers designed according to the sequence of *SQS* genes in *M. truncatula* and *Arabidopsis* were used for amplification (Table S2). The PCR amplicons were purified after agarose gel (1%) separation and cloned into pEASY-T3 vector (TransGen Biotech Corporation, Beijing, China). Sequencing confirmed *MsSQS* was used for subcloning. The qRT-PCR analysis was performed using the SYBR Premix Ex Taq (TaKaRa, Dalian, China) was used on BIO-RAD CFX96TM Real-Time System (BioRad, Hercules, CA, USA). *β-actin* was used as to normalize the loading. Three biological replicates were conducted.

### 4.4. Bioinformatic Analysis of MsSQS

For BLAST of *SQS* homologous genes, *AtSQS1* (At4g34640) was used as query sequence to search against EnsemblPlants (http://plants.ensembl.org/index.html). TMHMM (server v2.0) (http://www.cbs.dtu.dk/services/TMHMM-2.0) and SWISSMODEL (http://swissmodel.expasy.org/) were used to analyze the secondary structure and the three-dimensional homologous modeling, respectively. MEGA version 6.0 [32] was used for phylogenetic analysis and DNAMAN version 7.0 (Lynnon Corporation, Quebec, Canada) for multiple sequence alignment.

### 4.5. Constructions and Alfalfa Transformation

For different constructs, the ORF of *MsSQS* was amplified using primer pairs fitting the corresponding vectors and the amplicons were sequenced for verification. For GFP-fused construct (pA7-MsSQS-GFP), pA7-GFP vector and the sequence confirmed fragment with *Xho* I and *Sal* I sites were digested by the two restriction enzymes, and ligation was performed after purification of the digested fragments. For protein expression in *E. coli*, *MsSQS* and *MsSQSΔC30,* a truncated *MsSQS* lacking the C-terminal peptide of 30 amino acid residues, were amplified individually, and subcloned separately into pEASY-Blunt-E2 (TransGen Biotech Corporation, Beijing, China). For overexpression construct (*pBI121-MsSQS*), the ORF of *MsSQS* and pBI-121 were digested with *Xba I* and *BamH I.* The two fragments were ligated after gel purification. The plasmid of *pBI121-MsSQS* was introduced into *Agrobacterium tumefaciens* strain GV3101 by electroporation. Transgenic alfalfa was obtained by performing transformation as described by Jiang et al. [38]

### 4.6. Protein Expression in Transient and Prokaryotic System

For transient expression of *35S::MsSQS-GFP* or *35S::GFP*, the plasmid was transformed into onion epidermal cells by particle bombardment (Helios Gene Gun System, Bio-Rad, USA). After incubation for 24 h at 25 °C, cells were observed and image was taken using confocal laser scanning microscopy (Olympus FV500, Tokyo, Japan). For prokaryotic expression, *pEASY-E2*-*MsSQS* and *pEASY-E2*-*MsSQSΔC30* were transformed into *E. coli* Transetta (DE3) cells. Cells were treated with 0.8 mM IPTG at 30 °C for 5 h and proteins were extracted in buffer (50 mM Tris-HCl, pH 7.5, 10% glycerol, 5 mM DTT) as crude proteins from total cells. For proteins from supernatant, cell extraction was centrifuged at 12,000× *g* for 30 min at 4 °C, and the supernatant was collected. Boiled samples were separated on 10% SDS-PAGE and the gel was stained with Coomassie Brilliant Blue G-250, and de-stained gel (with solution of acetic acid:ethanol:H_2_O = 1:3:6) was imaged.

### 4.7. Measurement of the Content of Squalene Synthase Enzyme and Total Saponins

A plant squalene synthase kit (Crystalgen NingBo Biotech LTD, NingBo, China) was used to measure the content of squalene synthase, based on enzyme-linked immunosorbent assay (ELISA) technique, Leaf samples were measured according to the manufacturer’s instructions. Three biological assays were conducted independently. For measurement of the content of total saponins, leaf samples were used to extract total saponins according to the method described previously [50]. The content was measured with a spectrophotometer at a wavelength of 545 nm.

## 5. Conclusions

In this study, a squalene synthase (SQS) encoding gene *MsSQS* was isolated and characterized in alfalfa, an important legume forage worldwide. The deduced MsSQS possesses the main functional domains of SQS in eukaryotes and shares conserved exon/intron boundaries with its orthologs in higher plant. The ubiquitous *MsSQS* was expressed preferentially in roots relative to leaves and stems. *MsSQS* was up-regulated by MeJA and the treatment increased the content of MsSQS. Overexpression of *MsSQS* in alfalfa significantly enhanced the amount of saponins in an *MsSQS*-dependent way, indicating that the novel alfalfa *MsSQS* functions positively in saponin synthesis.

## Figures and Tables

**Table 1:** Comparison of the exon/intron composition of *SQS* in the indicated species.

Gene ID	Exon 1-0 ^a^	Exon 1	Exon 2	Exon 3	Exon 4	Exon 5	Exon 6	Exon 7	Exon 8	Exon 9	Exon 10	Exon 11	Exon 12	Exon 13
MsSQS		216	43	90	76	70	144	105	147	76	89	92	46	245
Mt4g071520		572	43	90	76	70	144	105	147	76	89	92	46	766
At4g34640		495	43	90	76	70	150 ^b^	105	147	76	89	92	43	426
At3g34650		330	43	90	76	70	150 ^b^	105	147	76	89	92	43	345
Gm12g038200		421	43	90	76	70	144	105	147	76	89	92	46	452
Gm11g112000		453	43	90	76	70	144	105	147	76	89	92	46	467
A4A49_02417		407	43	90	76	70	144	105	147	76	89	92	46	610
A4A49_25438		305	43	90	76	70	144	105	147	76	89	92	46	70
A4A49_10777		257	43	90	76	70	144	105	147	76	89	86	28	70
A4A49_12750		243	43	90	76	70	144	105	147	76	89	92	43	67
Os03g0805100		231	43	90	76	70	144	105	147	76	89	92	40	107
Os07g0200700		310	43	90	76	70	144	105	147	76	89	92	40	251
Zm00001d013048		320	43	90	76	70	144	105	147	76	89	92	40	106
Zm00001d034516		336	43	90	76	70	144	105	147	76	89	92	40	109
Pt009g123100v3		408	43	90	76	70	144	105	147	76	89	92	46	923
Pt004g161200v3	37	347	43	90	76	70	144	105	147	76	89	92	46	431
Ta5A02g454500		375	43	90	76	70	144	105	147	76	89	92	43	104
Ta5B02g464600		594	43	90	76	70	144	105	147	76	89	92	43	109
Ta5D02g465000		713	43	90	76	70	144	105	147	76	89	92	43	113
			√	√	√	√		√	√	√	√	√		
Cr03g175250v5		151	95	162	163	79	101	132	148	110	96	1208		
AAP36671.1		439	173	98	184	129	192	177	153	922				

^a^ an additional exon of populus *SQS*. ^b^ *Arabidopsis SQS* genes differ from other plants. √ represents the identical size of the exons in the indicated plants.

## Supplementary Materials

Supplementary materials can be found at https://www.mdpi.com/1422-0067/20/18/4499/s1.
