# Supplementary material for: Molecular Cloning and Functional Identification of a Squalene Synthase Encoding Gene from Alfalfa (Medicago sativa L.)
Source: Int J Mol Sci. 2019 Sep 11;20(18):4499. doi: 10.3390/ijms20184499 (PMC6770234; doi:10.3390/ijms20184499)
Supplement: Supplementary file 1 [file ijms-20-04499-s001.pdf]

Table S1. Sequence homology of SQS proteins in the indicated species.

Gene	Homology							
**Yeast**	100%							
**Human**	47.5%	100%						
**Chlamy**	39.2%	46.5%	100%					
**OsSQS1**	41.7%	48.1%	54.8%	100%				
**AtSQS1**	41.7%	45.9%	54.8%	72.6%	100%			
**GmSQS**	43.0%	48.1%	56.7%	79.0%	81.8%	100%		
**MtSQS**	42.0%	48.4%	56.7%	77.1%	79.0%	91.4%	100%	
**MsSQS**	41.4%	48.7%	57.0%	76.8%	79.0%	92.0%	97.8%	100%

Table S2. Sequence of primers used in this study.

Primer name	Sequence	Application
MsSQS-f	5′-ATGGGAAGywTrArwrCGATTTTG-3′	ORF amplification
MsSQS-r	5′- TTAkywmTTryrwyGATTkGCwkwGAGA -3′	
SQS-PE-f	5′-ATGGGAAGTATAAAAGCGATTTTG-3′	Prokaryotic expression
SQS-PE-r	5′-GTTATTGTAACGGTTGGCAGAGAG-3′	
SQS-PE-ΔC30-r	5′-CCTCAAAACGTAAGATTTCCTTTTGG-3′	
SQS-pA7-f	5′-CCCTCGAGATGGGAAGTATAAAAGCGATTTTGA-3′	subcellular localization
SQS-pA7-r	5′-GCGTCGACGTTATTGTAACGGTTGGCAGAGAGA-3′	
SQS-QPCR-f	5′-CTTCGGTCTTGTTATTCAGCAGC-3′	PCR/qRT-PCR
SQS-QPCR-r	5′-CTTGTATCATCCTCAACGGTGTC-3′	
Actin-f	5′-CAAAAGATGGCAGATGCTGAGGAT-3′	house-keeping gene
Actin-r	5′-CATGACACCAGTATGACGAGGTCG-3′	
SQS-f	5′-GCTCTAGAATGGGAAGTATAAAAGCGATTTTGA-3′	overexpression
SQS-r	5′-CGGGATCCTTAGTTATTGTAACGGTTGGCAGAG-3′	
35S-f	5′-ACTATCCTTCGCAAGACCCTTCCTC-3′	Identification of transgenic lines
SQS-r	5′-CGGGATCCTTAGTTATTGTAACGGTTGGCAGAG-3′	
gSQS-f1	5′-CTGAAAATCGCGGCGAGAAA-3′	Amplification of genomic sequence of *MsSQS*
gSQS-r1	5′-AGCTTTGATAAACCCAGCCCA-3′	
gSQS-f2	5′-GGACTTGTTGGGCTGGGTTT-3′	
gSQS-r2	5′-TTGTAACGGTTGGCAGAGAG-3′	
5′ -R1	5′-GCTGCTGAATAACAAGACCGA-3′	
5′ -R2	5′-ATCTGCTTCTCGGCGTTTCT-3′	
g-degenerate	5′-NTCGA(G/C)T(A/T)T(G/C)G(A/T)GAA-3′	
3′-f1	5′-GCTTATCTCTCTGCCAACCGT-3′	

Note: restriction site of *Xho I, Sal I, Xba* I and *Bam* H I was underlined.

Table S3. Protein property of SQS enzymes in the 12 indicated species.

Species	gene ID	cDNA (bp)	a.a	kDa	pI
M. Sativa	MsSQS	1439	413	47.25	7.53
M. truncatula	Mt4g071520	2316	413	47.08	7.71
A. thaliana	At4g34640	1902	410	47.14	6.59
	At3g34650	1656	413	47.18	8.01
G. max	Gm12g038200	1851	413	47.11	6.57
	Gm11g112000	1898	413	47.01	6.58
N. tabacum	A4A49_02417	1995	411	46.98	7.86
	A4A49_25438	1353	411	47.06	8.40
	A4A49_10777	1281	403	46.04	7.25
	A4A49_12750	1285	405	47.34	7.25
P. trichocarpa	Pt009g123100v3	2309	413	47.31	7.29
	Pt004g161200v3	1793	476	54.69	8.88
O. sativa	Os03g0805100	1310	403	46.10	7.86
	Os07g0200700	1533	410	46.78	6.97
Z. Mays	Zm00001d013048	1398	401	46.04	6.77
	Zm00001d034516	1417	403	46.18	7.06
T. aestivum	Ta5A02g454500	1706	404	46.35	7.04
	Ta5B02g464600	2123	404	46.49	7.04
	Ta5D02g465000	2327	404	46.38	6.76
C. reinhardt	Cr03g175250v5	2445	461	52.04	6.77
H. sapiens	AAP36671.1	2467	417	48.10	6.52
S. Cerevisiae	ACD03847.1	1350	444	51.73	5.74
**Average**		1774	416	47.68	7.20

Note: information on gene accession number is listed in the legend of Figure 1b.

Table S4. Prediction of the secondary structure of SQS in the indicated species.

Gene ID	alpha helix	random coil	beta turn	extended strand
**MsSQS**	69.25%	22.52%	4.12%	4.12%
**Mt4g071520**	66.34%	22.52%	4.36%	6.78%
**At4g34640**	70.70%	18.03%	4.23%	7.04%
**At3g34650**	68.97%	20.00%	4.48%	6.55%
**Gm12g038200**	67.31%	24.46%	3.15%	5.08%
**Gm11g112000**	69.49%	22.28%	3.87%	4.36%
**A4A49_02417**	69.10%	20.68%	3.65%	6.57%
**A4A49_25438**	68.61%	21.65%	3.65%	6.08%
**A4A49_10777**	69.73%	23.33%	3.47%	3.47%
**A4A49_12750**	66.17%	21.98%	3.46%	8.40%
**Os03g0805100**	70.22%	19.11%	5.21%	5.46%
**Os07g0200700**	70.00%	20.49%	4.39%	5.12%
**Zm00001d013048**	67.78%	24.92%	2.74%	4.56%
**Zm00001d034516**	68.73%	23.82%	3.97%	3.47%
**Pt009g123100v3**	68.52%	22.03%	3.63%	5.81%
**Ta5A02g454500**	71.04%	20.05%	3.71%	5.20%
**Ta5B02g464600**	67.82%	21.78%	4.21%	6.19%
**Ta5D02g465000**	67.82%	22.03%	4.46%	5.69%
**AAP36671.1**	67.87%	24.22%	3.12%	4.80%
**ACD03847.1**	67.79%	24.10%	2.70%	5.41%
**Average**	68.66%	22.00%	3.83%	5.51%

information on gene accession number is listed in the legend of Figure 1b.

```
                                                                 GCTTATTCGTAGAAACAAAAG
1     ATGGGAAGTATAAAAGCGATTTTGAAGAATCCAGATGATTTTTTTCCATTACTTAAGCTGAAAATCGCGGCGAGAAAC
1      M  G  S  I  K  A  I  L  K  N  P  D  D  F  F  P  L  L  K  L  K  I  A  A  R  N

79    GCCGAGAAGCAGATCCCACCGGAGCCGCATTGGGGATTCTGTTACTCTATGCTTCATAAGGTTTCTAGAAGCTTCGGT
27     A  E  K  Q  I  P  P  E  P  H  W  G  F  C  Y  S  M  L  H  K  V  S  R  S  F  G

157   CTTGTTATTCAGCAGCTTGGTCCCGAGCTTCGTGATGCTGTTTGCATATTCTATTTGGTTCTTCGTGCTCTTGACACC
53     L  V  I  Q  Q  L  G  P  E  L  R  D  A  V  C  I  F  Y  L  V  L  R  A  L  D  T

235   GTTGAGGATGATACAAGCATAGAAACAGATGTCAAGGTTCCCATACTAATGGATTTTCATCGTCACATCTATGATAAT
79     V  E  D  D  T  S  I  E  T  D  V  K  V  P  I  L  M  D  F  H  R  H  I  Y  D  N

313   GATTGGCACTTTGGGTGTGGCACGAAAGAGTACAAAGTTCTAATGGACCAGTTCCATCATGTTTCAAAGGCTTTTCTG
105    D  W  H  F  G  C  G  T  K  E  Y  K  V  L  M  D  Q  F  H  H  V  S  K  A  F  L

391   GAACTTGGAAAGAACTATCAGGATGCAATCGAGGACATTACGAAAAGAATGGGTGCTGGAATGGCGAAATTCATTTGC
131    E  L  G  K  N  Y  Q  D  A  I  E  D  I  T  K  R  M  G  A  G  M  A  K  F  I  C

469   AAGGAGGTAGAAACAATTGATGACTACGATGAATATTGTCACTATGTGGCTGGACTTGTTGGGCTGGGTTTATCAAAG
157    K  E  V  E  T  I  D  D  Y  D  E  Y  C  H  Y  V  A  G  L  V  G  L  G  L  S  K

547   CTTTTCTACGCATCTGGTAAAGAAGATCTGGCTACAGACAAACTTTCAAACTCAATGGGTTTGTTTCTTCAGAAAACC
183    L  F  Y  A  S  G  K  E  D  L  A  T  D  K  L  S  N  S  M  G  L  F  L  Q  K  T

625   AACATTATTCGAGATTATCTGGAAGACATCAATGAGATACCAAAGTCACGCATGTTTTGGCCACGGCAGATCTGGAGT
209    N  I  I  R  D  Y  L  E  D  I  N  E  I  P  K  S  R  M  F  W  P  R  Q  I  W  S

703   AAATATGTTATCAAACTTGAGGACTTGAAATATGAGGAAAACTCCGTTAAGGCTGTGCAATGCTTAAATGACATGATC
235    K  Y  V  I  K  L  E  D  L  K  Y  E  E  N  S  V  K  A  V  Q  C  L  N  D  M  I

781   ACTAATGCTTTGCTGCATGCTGAAGATTGCTTACAATACATGTCTGCATTACGAGACCCCTCTAATTTTCGCTTTTGT
261    T  N  A  L  L  H  A  E  D  C  L  Q  Y  M  S  A  L  R  D  P  S  N  F  R  F  C

859   GCTATTCCTCAGGTAATGGCAATTGGAACACTTGCAATGTGCTATAACAACATTGGTGTTTTCAGAGGAGTAGTTAAA
287    A  I  P  Q  V  M  A  I  G  T  L  A  M  C  Y  N  N  I  G  V  F  R  G  V  V  K

937   ATGAGGCGAGGTCTAACTGCCAAAGTGATTGACCGAACAAAGACTATGGCTGATGTCTATGGTGCTTTCTTTGATTTT
313    M  R  R  G  L  T  A  K  V  I  D  R  T  K  T  M  A  D  V  Y  G  A  F  F  D  F

1015  GCTTCCATGTTGGAGTCCAAGGTTGACAAAAATGATCCAAATGCAACAAAAACATCGAGCAGGCTGGAAGCTATACAG
339    A  S  M  L  E  S  K  V  D  K  N  D  P  N  A  T  K  T  S  S  R  L  E  A  I  Q

1093  AAAACTTGCAGAGAATCTGGTCTCCTAACCAAAAGGAAATCTTACGTTTTGAGGAATGAGAGCGCATATGGCTCTACC
365    K  T  C  R  E  S  G  L  L  T  K  R  K  S  Y  V  L  R  N  E  S  A  Y  G  S  T

1171  ATGATTCTCTTACTGGTCATCTTGTTGTCCATCTTTTTTGCTTATCTCTCTGCCAACCGTTACAATAACTAA
391    M  I  L  L  L  V  I  L  L  S  I  F  F  A  Y  L  S  A  N  R  Y  N  N  *
      TGTAAGTTCTATTTAATTTTGTTTTTGGTAAACAGTGTTTTGAATGTATGCTAGAATGTGTGAGAAATTAACAAAATACT
      AAGTTATCCTTTCTGATGAAATAGTTCAATTCTTTTTTGTAGCAACGGGTATTATGGCCTTCACAAACATGGATAGCAA
      CAGAACAGTGGTAGCAT
```

Figure S1. cDNA sequence of *SQS* cloned from alfalfa and the deduced protein sequence. The ORF was numbered and * represents the stop code (TAA).

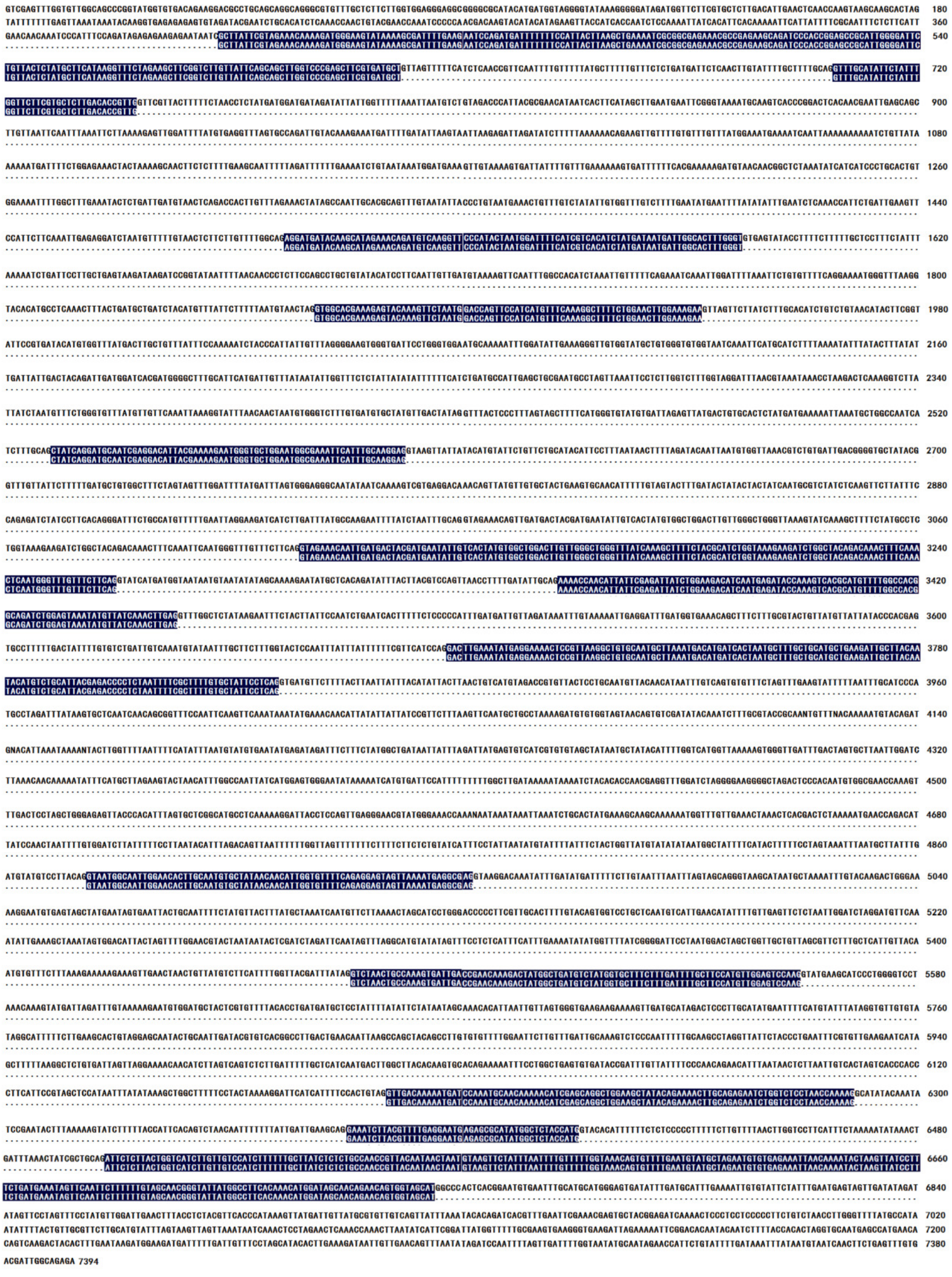

Figure S2. Squence alignment of *MsSQS* showing exon-intron composition.

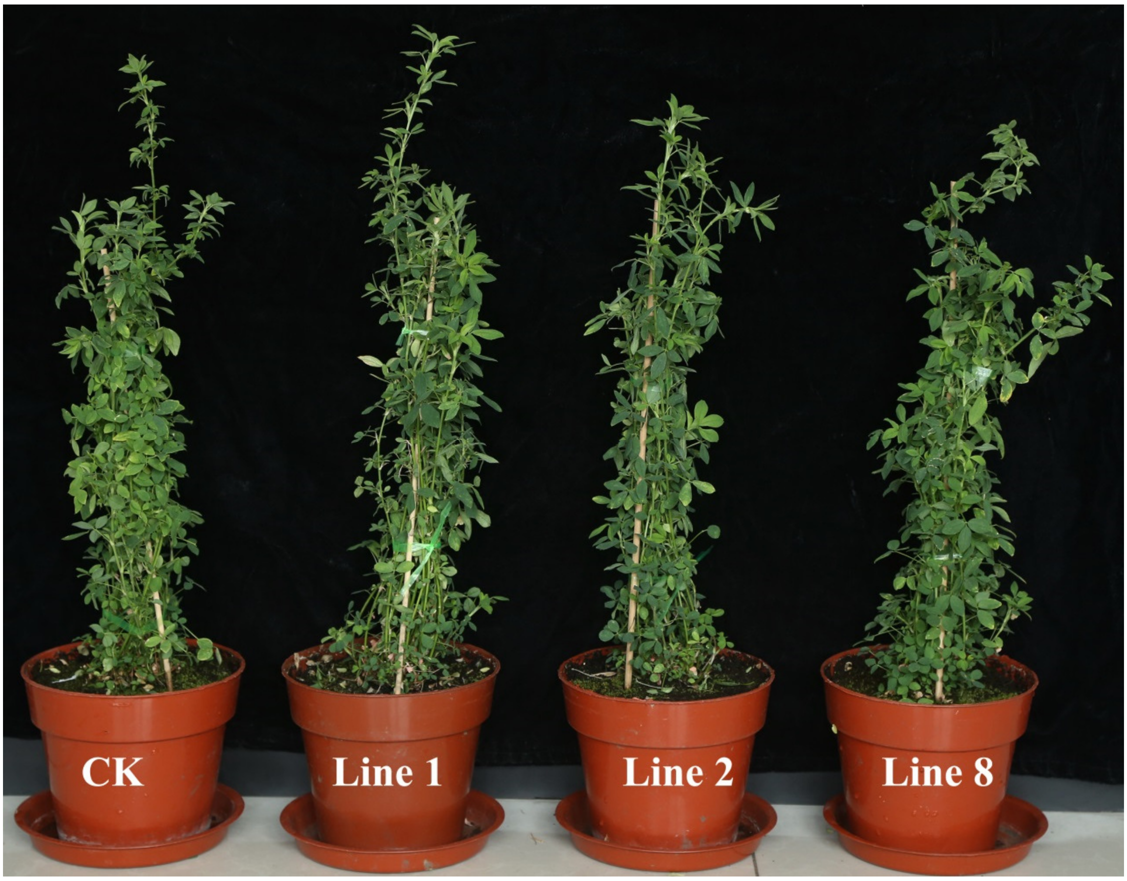

Figure S3. Image of 3-month-old control and the three transgenic alfalfa lines overexpressing *MsSQS*.
